# Reduction in Fall Rate in Dementia Managed Care Through Video Incident Review: Pilot Study

**DOI:** 10.2196/jmir.8095

**Published:** 2017-10-17

**Authors:** Eleonore Bayen, Julien Jacquemot, George Netscher, Pulkit Agrawal, Lynn Tabb Noyce, Alexandre Bayen

**Affiliations:** ^1^ Pitie-Salpetriere Hospital - Assistance Publique Hôpitaux de Paris (APHP) & University Pierre et Marie Curie Department of Neuro-Rehabilitation Global Brain Health Institute, Memory and Aging Center, University of California, San Francisco Paris France; ^2^ SafelyYou Inc. at SkyDeck (Chief Technology Officer) Electrical Engineering and Computer Sciences University of California Berkeley, CA United States; ^3^ SafelyYou Inc. at SkyDeck (Chief Architect) Electrical Engineering and Computer Sciences University of California Berkeley, CA United States; ^4^ Kentfield Hospital Kentfield, CA United States; ^5^ Center for Information Technology Research in the Interest of Society and SafelyYou Inc. at SkyDeck (Chief Scientist) Electrical Engineering and Computer Sciences University of California Berkeley, CA United States

**Keywords:** video monitoring, video review, mobile app, deep learning, fall, Alzheimer disease, dementia

## Abstract

**Background:**

Falls of individuals with dementia are frequent, dangerous, and costly. Early detection and access to the history of a fall is crucial for efficient care and secondary prevention in cognitively impaired individuals. However, most falls remain unwitnessed events. Furthermore, understanding why and how a fall occurred is a challenge. Video capture and secure transmission of real-world falls thus stands as a promising assistive tool.

**Objective:**

The objective of this study was to analyze how continuous video monitoring and review of falls of individuals with dementia can support better quality of care.

**Methods:**

A pilot observational study (July-September 2016) was carried out in a Californian memory care facility. Falls were video-captured (24×7), thanks to 43 wall-mounted cameras (deployed in all common areas and in 10 out of 40 private bedrooms of consenting residents and families). Video review was provided to facility staff, thanks to a customized mobile device app. The outcome measures were the count of residents’ falls happening in the video-covered areas, the acceptability of video recording, the analysis of video review, and video replay possibilities for care practice.

**Results:**

Over 3 months, 16 falls were video-captured. A drop in fall rate was observed in the last month of the study. Acceptability was good. Video review enabled screening for the severity of falls and fall-related injuries. Video replay enabled identifying cognitive-behavioral deficiencies and environmental circumstances contributing to the fall. This allowed for secondary prevention in high-risk multi-faller individuals and for updated facility care policies regarding a safer living environment for all residents.

**Conclusions:**

Video monitoring offers high potential to support conventional care in memory care facilities.

## Introduction

A fall is defined as an “unexpected event in which the participant comes to rest on the ground, floor, or lower level” [1]. Falls are the leading cause of both fatal and nonfatal injuries among people aged 65 and older, with estimated yearly direct medical costs of US $637.2 million for fatal falls and US $31.3 billion for nonfatal falls in the United States alone [2]. Incidence of falls in people with cognitive impairment is estimated to be twice that of cognitively intact older adults [3]. In nursing facilities, individuals with dementia fall 4.05 times per year on average versus 2.33 times per year for other residents [4]. Fall accidents represent the primary cause of Alzheimer disease–related hospitalizations, contributing to 26% of all hospitalizations in the United States [5].

Detecting a fall early and in an ongoing manner provides significant potential for reduced morbidity and mortality in patients and system-wide savings [6]. As 50% to 75% of elderly fallers experience recurrent falls [7-11], detecting the first fall and taking preventative action provides significant potential for reducing fall risk, fall-related injuries, and fall consequences at large [12]. A rapid detection of fall limits the long-lie (ie, the amount of time fallers spend lying on the ground), which has been shown to be a predictor of worse independent walking capacity and autonomy and longer length of hospitalization [10,13]. Real-time diagnosis of falls might result in a more accurate identification and care of direct fall-related injuries (eg, traumatic brain injury and orthopedic fractures) and in lowering short-term indirect consequences (eg, pressure sore, hypothermia, and phlebitis) as well as long-term fall-related consequences (eg, fear of falling again, loss of autonomy as a result of postfall restrictions, and social isolation) [14,15]. As a consequence, considerable research about fall prevention [16] has been conducted with a higher level of evidence for environmental modifications in the homes [17], management of symptomatic hypotension and depression [18], exercise programs in mobile seniors, and combined supplementation of vitamin D and calcium [19,20]. Over the past years, fall management has also become a key criterion of quality of care worldwide and in care facilities in particular [12,15,21-23].

A significant portion of recent health technology innovation regarding fall management has been driven by industry and has taken place in the commercial space. To date, the most well-known commercial solutions include wearable alert systems [24], which demonstrate limited success in dementia care because individuals forget or refuse to wear a device; nonwearable fall detection systems, which are based on radar and optical sensors, are under development but not commercially available in the United States yet [25] and have not demonstrated robustness through evidence-based medical studies [26]; fall mats and bed alarms, which are prevalent solutions in memory care [27] but suffer from high false alarm rates and are mainly targeting those residents who should never be walking independently; and accelerometer-based fall detection [28], which provides meaningful information about the biomechanical features of fall but fails to give a holistic and clinically useful picture of falls (including assessment of environmental hazards). Overall, none of these strategies allow care providers to identify globally how and why a fall occurs and thus leverage this information to enhance safety in residents and improve quality of care practice in the facility staff.

In this study, the video technology was used to review real-world falls in a single memory care facility, thus avoiding artificiality of simulated or acted falls carried out in a contained laboratory environment, as well as biased information about falls gathered from individuals’ recalling the fall or from administrative hospital record [29]. The extent to which video monitoring and fall review can impact quality of care practice and health outcomes is in fact a relatively new and unexplored field. The most relevant work on video monitoring of falls has been conducted by Robinovitch et al [30-33]. In part of that work, video recording was collected from cameras installed in common spaces of two Canadian long-term care facilities in charge of elderly residents over a period of 3 years. In a dataset of 227 falls captured for 130 individuals, the authors confirmed an increased fall incidence among residents with Alzheimer disease and identified the most frequent fall mechanisms in managed care facilities, including incorrect weight shifting (41%), trip or stumble (21%), hit or bump (11%), loss of support (11%), and collapse (11%) [30]. However, the video review process was not carried out with facility staff with the specific intention of identifying and removing any possible causes or providing obvious changes to the environment that staff could address. Another study conducting video monitoring recorded 25 falls in 17 elderly subjects in the lobby of a geriatric complex over 15 months. This group identified predominant causes of falls, including intrinsic factors (60%), environmental factors (36%), and behavioral factors (4%) but did not report any interaction with medical and paramedical staff either [34]. Thus, previous work in the field offers little insight into the effect of introducing cameras and how video review can impact fall rate and care practice.

A holistic approach of the fall management was used in this paper. The objective of the study was to analyze how continuous video monitoring and video review of falls occurring in common spaces and private rooms of residents living in a memory care facility can support best quality of care.

## Methods

### Design of the Study and Population

This study reports on an ancillary study that is part of a larger project called SafelyYou. SafelyYou aims at developing deep learning (a subfield of machine learning) algorithms for automated real-life real-time fall detection in nursing and memory care facilities (http://www.safely-you.com). This pilot observational study was carried out between July and September 2016. Falls were video-captured in residents 24 hours a day, 7 days a week, and the video recordings were provided to the facility staff for video review. The study took place in a memory care facility that is part of the Memory Care Community in California and of the Integral Senior Living network, in which residents reside in a supportive ecosystem. The facility offers 40 individual bedrooms with individual bathrooms and common indoor areas (2 living rooms, 2 eating areas, and kitchens and hallways) where residents are allowed to walk and spend time freely. Residents of this memory care facility have all been diagnosed with dementia (Alzheimer disease and related dementias), had a mean age of 79.4 years (standard deviation [SD] 3.2), and were predominantly female (71.4%) at the time of the study inclusion.

### Outcome Measures

The primary outcome measure is the count of the total number of residents’ falls occurring in the video-covered areas of the facility over the 3-month period of video recording (allowing us to compute a fall rate per month). This count is further compared with the cases of falls that the facility health board independently reported in its daily routine care for each known occurrence of fall (ie, administrative report regardless of the video recording) 2 months before video deployment (baseline occurrence, May-June 2016) and during the 3 months of study (July-September 2016).

The secondary outcome measures qualitatively assess the use of video recording and replay possibilities for care practice. This entails (1) acceptability of video monitoring by residents and facility staff and use of fall review by facility staff to support care practice and quality of care; and (2) the analysis of falls and of fall-related injuries, leveraging video replay to depict intrinsic and extrinsic factors, and environmental circumstances contributing to the falls Acceptability and impact of video review on care practice were assessed through semidirected interviews carried out during bimonthly meetings with the care facility staff over the 3 months of study. An adapted version of the 4-point Hopkins Falls Grading Scale [35] was used to stratify fall severity in near-fall (Grade 1), fall with no need for medical examination (Grade 2), fall requiring medical attention (Grade 3), and fall requiring hospital admission (Grade 4). The fall events were also classified using the International Classification of Disease, Tenth Edition (ICD-10) published by the World Health Organization [36]. A description of what could be identified as cognitive-behavioral dysfunction by itself and as a response to the social-contextual stimuli of the living environment around the individual leveraging to video recording just before and during the fall was provided.

### Equipment and Process

**Figure 1:**
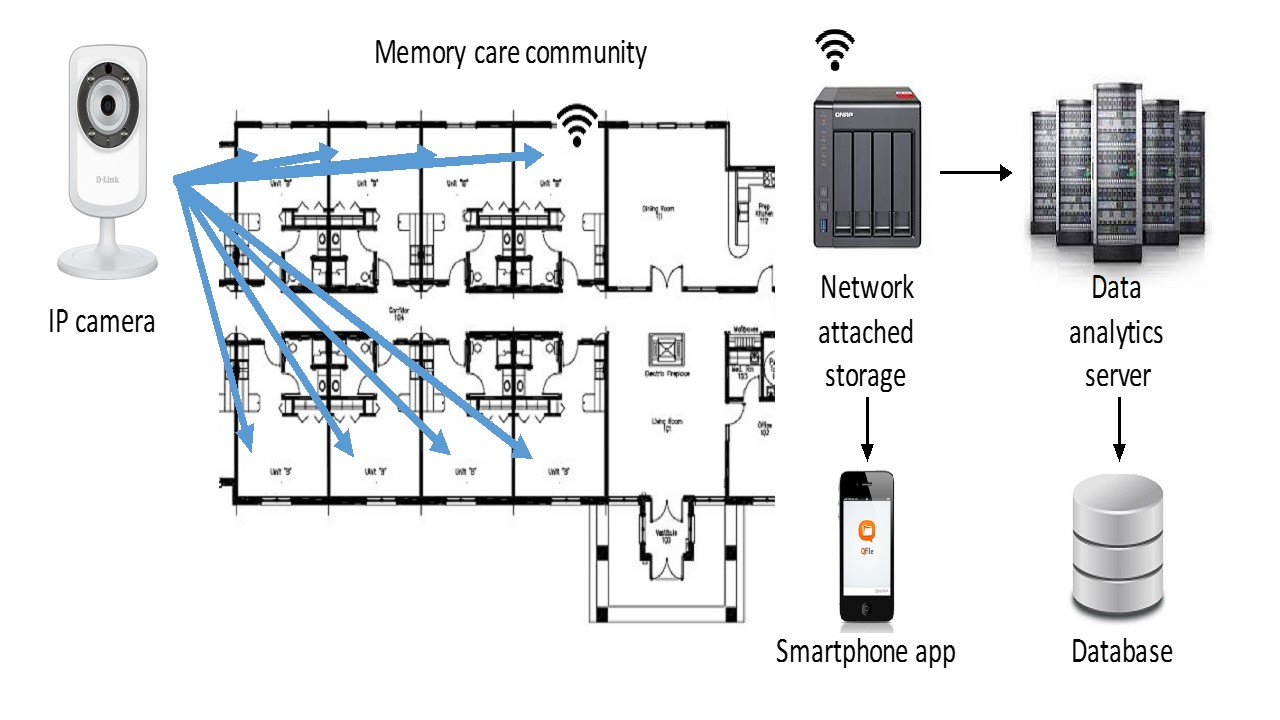
Loop equipment, including Internet Protocol (IP) cameras, network attached storage, Wi-Fi, secured storage on the university server, and phone apps.

A total of 43 wall-mounted cameras were deployed in all common areas and private rooms of consenting residents and families in accordance with the following privacy and ethical guidelines. Figure 1 shows the off-the-shelf video-recording equipment used. Video data were transmitted using Wi-Fi to local network attached storage (NAS) devices. Facility Wi-Fi coverage was upgraded using off-the-shelf routers and range extenders to remove Wi-Fi dead zones. Video was maintained on the local NAS for 72 hours before transmitting to a university server where the complete video dataset was maintained encrypted on a password-protected server. A customized mobile device app was provided for viewing video from the previous 72 hours, developed by the makers of the NAS. The mobile device app for accessing the live video from each camera was provided as developed by the camera manufacturers. Cameras were configured to only record motion and to filter unneeded video. Software was developed to support video transcoding and uploading from the NAS to work around bandwidth limitations defined by the upload speed granted to the memory care facility through their Internet service provider. The specific equipment provided to the facility included the following: 43 DLink 932L IP camera, 2 QNAP 451 including network attached storage, 2 Netgear AC5300 Nighthawk X8 WiFi Router, and 2 Netgear Nighthawk AC1900 WiFi Range Extender. Data were securely stored. The research team had access to the data through a password-protected computer in locked laboratories that are part of virtual private networks.

The videos of fall events that had been depicted by the research team were made available to be viewed by the executive director of the facility who would decide to discuss them with her staff. The meetings between the facility staff and research team were carried out twice a month during the 3 months of the study in a rather flexible way and using semidirected interviews. The main purposes of these meetings were as follows: (1) to be sure that no unanticipated issues or concerns with residents, surrogates, and/or staff arose and (2) to observe the use (or no use) of the videos and what were the changes in care practice that were reported. During these meetings, the research team asked about the use of the videos in a neutral way (ie, observing the potential uptake of the recording without pushing attitude). The main focus of the first meeting concerned the confirmation of the resident-surrogate dyads who had agreed to participate, as well as the questions from the executive director. The final meeting focused on the removal of all the cameras of the facility and discussed the practice changes that the video recording had potentially triggered.

### Ethical Procedures and Privacy Concerns

Privacy and consent procedures were developed with support from the institutional review board (IRB) of the University of California, Berkeley (http://cphs.berkeley.edu/), and following guidelines from California Department of Social Services Community Care Licensing Division (CDSS-CCLD). Approval of the study protocol was obtained from the Committee for Protection of Human Subjects of University of California, Berkeley, before starting the study (CPHS protocol number 2015-11-8119). Residents living within the care facility showed severe cognitive impairment related to Alzheimer disease and related dementias. Their capacity to consent to research according to the legal standards of informed consent was altered. As a consequence, surrogate consent was required for this pilot study. The legally authorized representatives of the facility residents were informed at a town hall meeting that a study on fall prevention would occur at the facility and were invited to participate in its presentation with their relative. The legally authorized representatives of the facility residents were given oral and written information about the purpose of the study, procedures, risks, and benefits as listed in the consent form. Those who would like to participate signed the self-certification document to confirm they were the legally authorized representatives and were provided the informed consent document provided by the research team. The study was explained to the affected individuals living in the facility. If affected individuals provided assent, they would be included in the study. If they provided any verbal or nonverbal indication that they do not wish to have the camera in their room or object to any other part of the study, they would not be included. The legally authorized representative was the one who could say yes to the study, thus providing informed consent, but the resident retained the right to say no to the study at any time, thus providing assent. If at any time, individuals expressed verbal or nonverbal indication that they would like the camera removed, personnel would remove the cameras. Participants or legally authorized representatives who originally assented or consented to the study and would later revoke consent would also have cameras removed and video data destroyed.

In private bedrooms, cameras were located high-up in a corner in the bedroom but not in the bathroom and remained visible to the participants. When cameras were not unplugged, they would show a small red light when motion is detected in a room. A sticker was positioned on the participants’ doors as a reminder to the residents, families, and facility staff that participants were being filmed in their private rooms. This physical sign on the door stating that video recording was in progress ensured that everyone entering the room was aware of the camera. Flyers that explained the goals of the research study, the length of the study, the use of wall-mounted cameras, and the generic email address and centralized phone number were positioned in several locations of the facility. The generic study email address and the centralized phone number were provided to respond to any withdrawal wish, expression of interest, or questions. Cameras were also equipped with an explanatory tag that described the goals of the research, the use of wall-mounted cameras, and the possibility to unplug the camera at any time and the way to do so, as well as the name of the principal investigator, the generic study email address, and the centralized phone number to be used in case of concerns. The guidelines from CDSS-CCLD were followed for the study protocol. Whereas the federal law requires that all residents have the right to privacy, the CDSS guidelines for use of the video surveillance state that recording in a common area does not require a waiver because there is no expectation of privacy in common areas (such as eating areas) [37]. Cameras were finally removed shortly after the end of the study.

A registered nurse was hired specifically for the study and was available to answer concerns from the participants, the families, and the facility staff, which could emerge before and during the study, including potential withdrawal from the study. If the participant or his/her legally authorized representative expressed willingness to withdraw from the study, they were to inform either the facility staff who would transmit this information to the nurse or the research team by directly using the generic study email address and/or the centralized phone number generated for the study. The possibility of participants’ withdrawal from the study at any point was mentioned at both oral and written levels during information and inclusion sessions. As mentioned on the camera laminated tag, the equipment could also be turned off at any time by simply unplugging it from the wall outlet. If the camera had been unplugged for over 24 hours, the team would figure out whether the participant or surrogate forgot to plug the camera back in or whether he/she would like to have the camera removed for the rest of the study. If a participant or his/her surrogate wished to withdraw the study at any time, all his/her video data would be destroyed. Video segments found improper by the review board were referred to the dementia care nurse of the team in case of content of potential physical or sexual abuse, neglect, sexual activity, or other actions that could imply abuse if taken out of context and other incriminating behaviors. Before deleting data, the dementia care nurse was responsible for determining whether the matter should be taken to facility management or to adult protective services. In accordance with Californian legislation [37], facility management granted permission to place cameras in common areas. Following California state guidelines [37], audio recording was disabled and signs were posted visibly on the door of each private room in which video recording occurred. Before publishing video or pictures in any way, signatures of individuals contained in the videos or their surrogate decision makers were obtained on media release written forms, allowing for public release of the specific videos in question. Faces were blurred on the video images to minimize identifiers in some cases.

## Results

### Participation and Acceptability of Video Monitoring

A total of 15 out of 38 resident-family dyads (40%) were able to attend the information meeting about the research study, out of which 10 gave oral and written consent and volunteered for the research, and 5 did not wish to participate. Accordingly, the video recording in private rooms included 10 residents, and video recording in common spaces included the total of 38 residents in July and August, followed by 36 residents in September (because of a slight dip in facility occupancy rate).

No impact of the video deployment, recording, and review on the daily routine of the residents and professional caregivers was reported over the 3-month period. At the end of the study period and based on the preliminary results and care experience, the project partner of memory care facilities of Integral Senior Living network agreed to expand the protocol to 14 facilities.

### Fall Review Utilization by Facility Staff

Bimonthly follow-up interviews showed that, in the first 7 weeks of the study, no formal video review was carried out by facility staff despite the fact that video recordings from the previous 72 hours were easily available through secured mobile devices to facility management. Facility management reported hardly ever using the video feeds during this time because of the numerous other challenges faced with operating a memory care facility and the little obvious value granted to the video so far. After 7 weeks, a particularly severe fall incident was recorded during daytime in which the resident was lying on the ground for almost 3 hours without receiving assistance. In accordance with procedures approved by the IRB of the university, this incident was reported to facility management. After reviewing this fall, facility management showed increased interest in reviewing other falls, and the mobile device app provided to review videos proved to be accessible and easy to use to facility staff, who subsequently gained familiarity with it. Further interviews revealed that facility management found video replay useful to grade the severity of the injury and eventually screen patients in the future for external referral to the emergency unit in case of severe injury. In addition, interviews revealed that facility management carried out preventative care interventions, which they believed would address some of the causes of future falls. These preventive actions first included moving furniture and changing room layout based on potential tripping hazards and falls (noticed from videos). Second, changes to care policy that included additional checking on high-risk residents every hour instead of every 2 hours at night were instated following the review of the data.

### Falls Count Over Facility Space and Study Period

**Figure 2:**
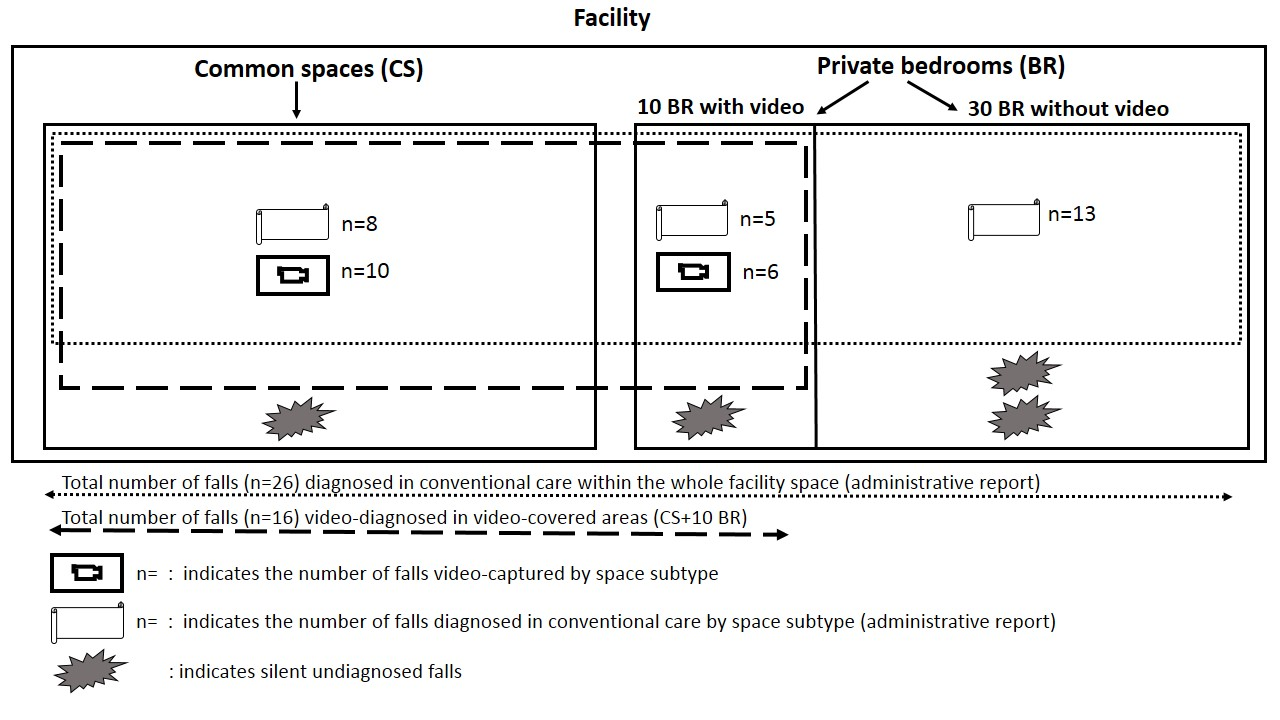
Fall count display over video-covered and video-uncovered areas.

During the 3-month intervention period, a total of 26 falls were reported in routine conventional care by facility staff for the whole facility (in both video-covered and video-uncovered areas; Figure 2). A total of 16 falls were video-captured and recorded in video-covered areas including 3 falls that were neither witnessed nor recorded by facility staff (Figure 2). In these 3 falls, the resident stood up alone after the fall (as shown in the pictures), and neither care nor administrative report was provided for these cases that would have remained silent falls if not video-witnessed. In other words, without the system, the falls and potential injuries would have gone unnoticed. Among these 16 video-captured falls, 10 happened in common spaces (in a single multi-faller woman) and 6 in private bedrooms (in 4 men fallers) (Table 1). The 13 video-uncaptured falls that were reported in conventional care happened in private rooms of individuals who had not volunteered for the research.

**Figure 3:**
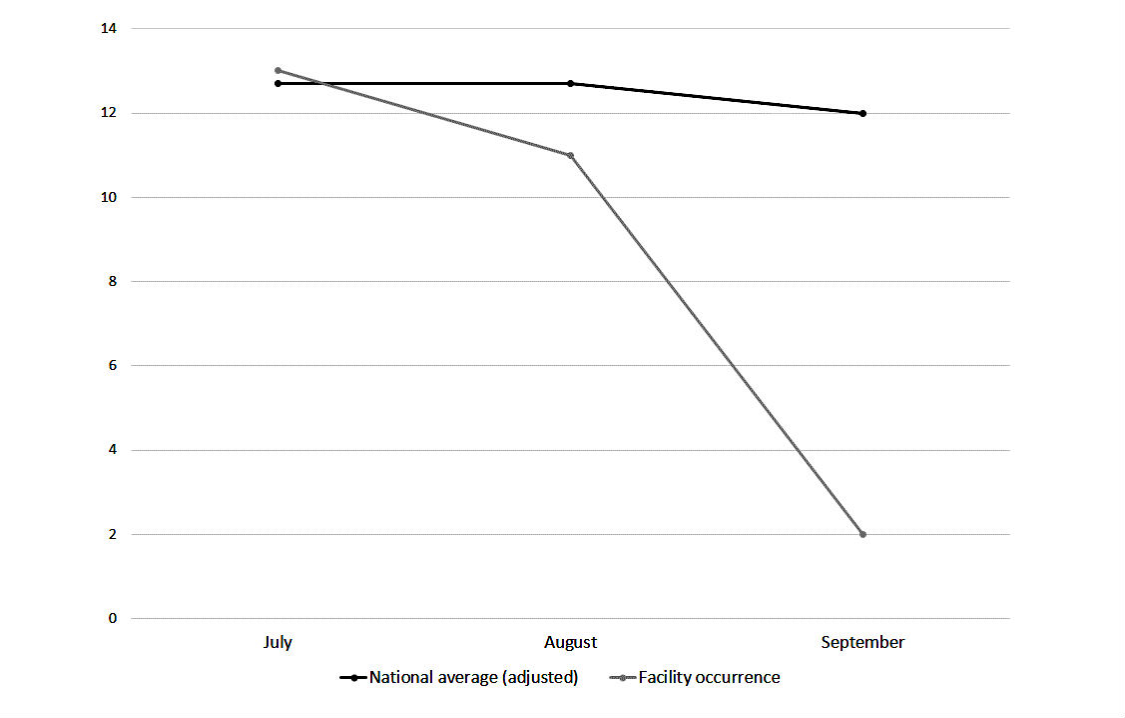
Fall rate per month displayed over the 3-month study period.

In the 2 months before the video deployment, a total of 18 falls were administratively reported (11 in May and 7 in June), providing a prevideo intervention facility baseline fall rate of a mean of 9 falls per month. An expected facility fall rate adjusted for the number of residents of 12.7 and 12 falls per month was reported for comparison purpose in Figure 3. The fall rate was shown to decrease over the 3-month period from a mean 12 falls per month (average in July and August) to 2 falls during the last month of the study, that is, September 2016. Figure 3 shows that the overall fall rate in this community was 79% of the national average for the 4 months before review and 17% of the national average in the month following review.

### Fall Review for Screening Fall Risk Patterns in Residents

**Figure 4:**
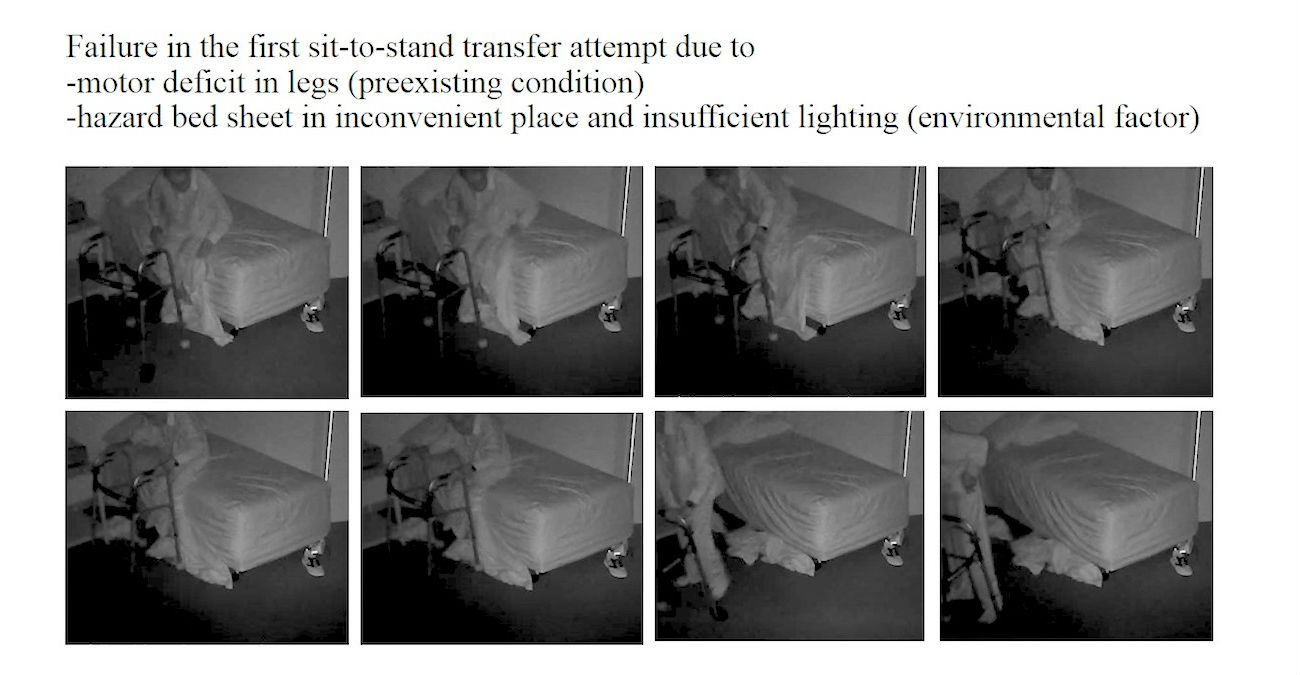
A video-witnessed pre-fall activity (subject 4, in his private bedroom). Reproduced with permission of the individual and his family.

**Figure 5:**
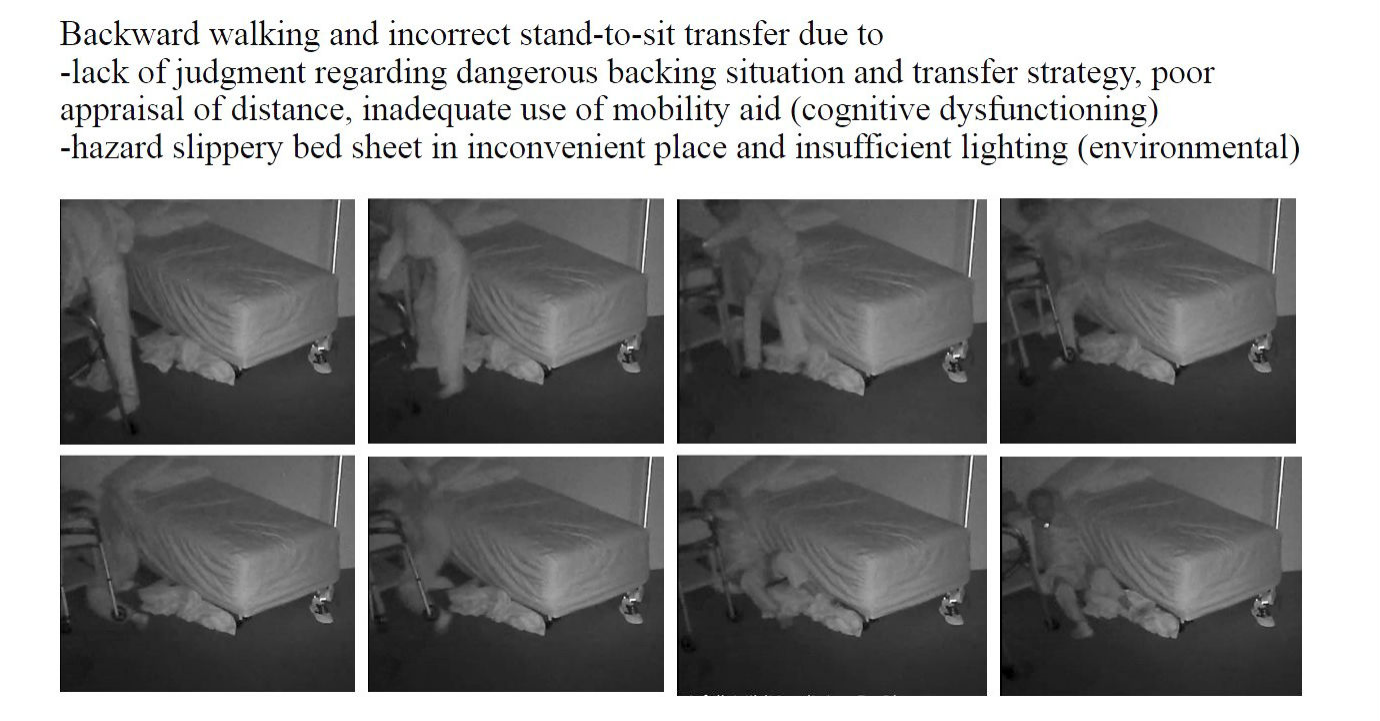
A video-witnessed backward fall event (subject 4, in his private bedroom). Reproduced with permission of the individual and his family.

**Figure 6:**
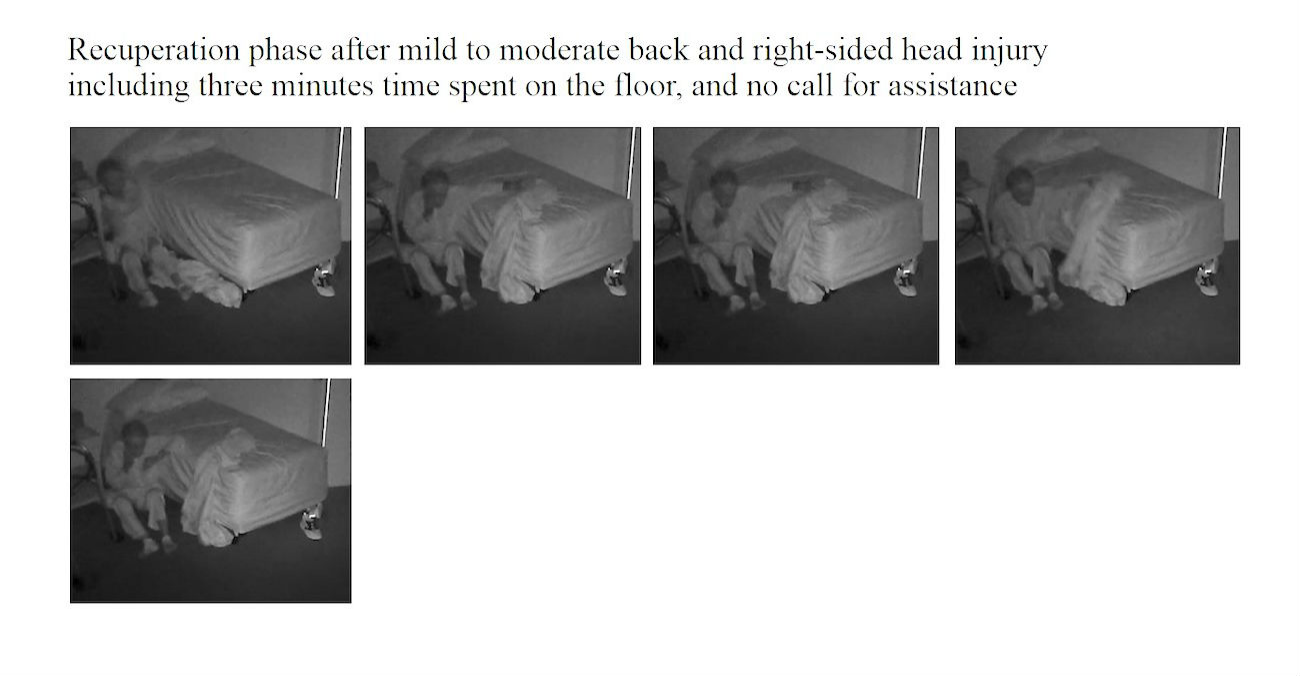
A video-witnessed post-fall recuperation (subject 4, in his private bedroom). Reproduced with permission of the individual and his family.

**Figure 7:**
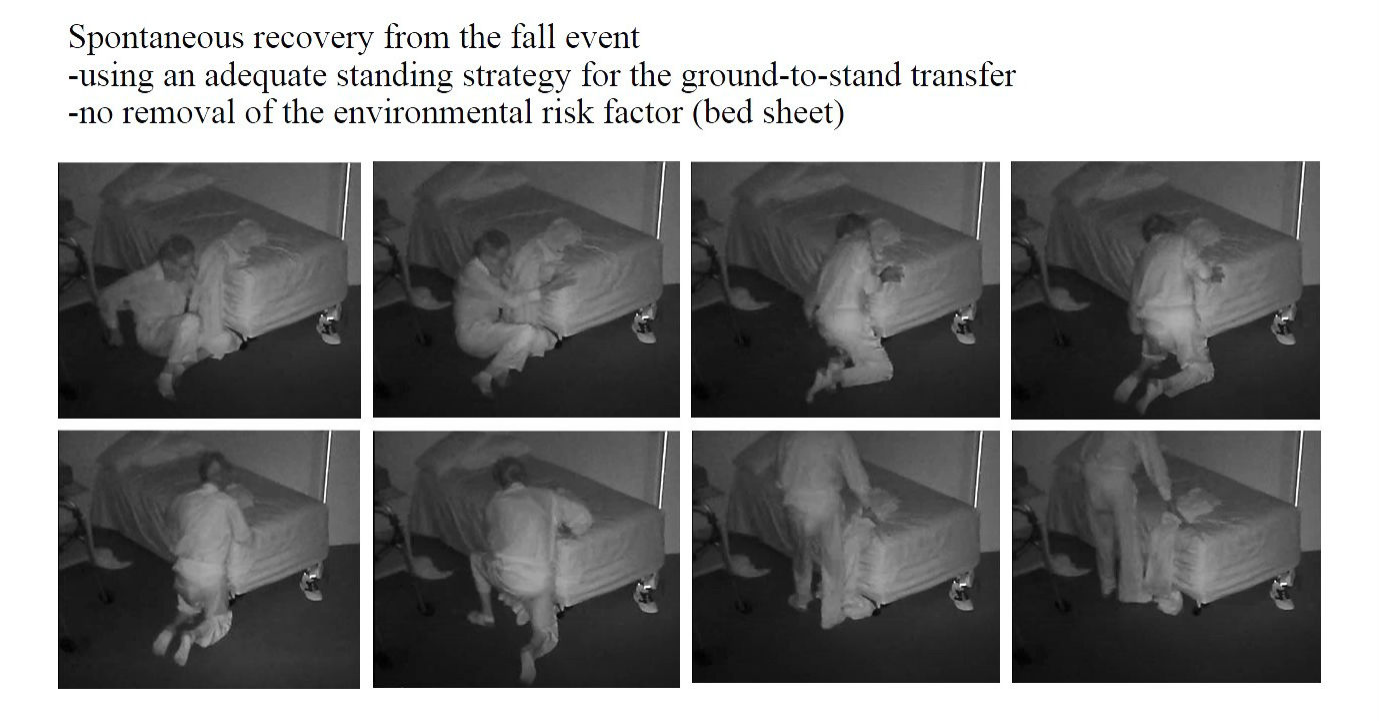
A video-witnessed post-fall activity (subject 4, in his private bedroom). Reproduced with permission of the individual and his family.

As summarized in Table 1,10 out of 16 (62%) falls happened in a multi-faller woman (subject 1), showing quite similar repetitive patterns of falls in common spaces during daytime. Conversely, 6 out of 16 falls (38%) occurred in the residents’ bedrooms and half occurred at nighttime. One resident had a moderate head injury (subject 4; Figures 4-7) but stood up alone, and the fall remained unnoticed by the facility. For the 3 other bedroom fallers (subjects 2, 3, 5), a routine diagnosis and report of falls were carried out, as all 3 residents were found lying on the ground. However, the circumstances and natural history of these falls remained unwitnessed and unknown to the staff until they retrospectively video-witnessed why and how the residents fell. Among these 16 falls, biomechanical causes related to preexisting conditions were identified in terms of incorrect shift of body weight, gait disturbances, loss of external support, or motor deficit in legs. According to the Hopkins scale, falls were, on average, moderately severe (mean 2.5; min 2, max 4), but 83% of bedroom falls would have required medical attention (Table 1). Falls occurred predominantly during transfer activities (63%; Table 1). As shown in Table 1 and Figures 4-7, understanding the interaction of the resident with his or her living environment just before, during, and after the fall revealed that extrinsic factors were contributing to the fall in all bedrooms. In addition, dysfunction of cognitive-behavioral processing could be assessed in terms of lack of judgment on self-deficits, poor awareness of dangerous transfer situations and of dual-tasking activities, over-reactivity to external distractors or inattention, and impulsivity (Table 1).

**Table 1:** Characteristics of falls in a sample of 16 falls collected in 5 individuals over a 3-month period.

Distribution				Severity			Fall circumstances		
Subject (S#)^a^and fall		Location	Time^b^	Body impact	Head injury	Severity grading	Activity performed (corresponding to ICD-10 code)^c^	Interaction with the living environment as a contributor to the fall: 1. extrinsic factor 2. cognitive-behavioral processing	Got up alone
**S1 F**									
	#1	CS^d^	D	0	0	2	Transfer sit-to-stand while talking (W07^e^)	No extrinsic factor identified Distraction/inattention in dual tasking (talking to caregiver when transferring)	0
	#2	CS	D	0	0	2	Slipping from chair (W07^e^)	No extrinsic factor identified	0
	#3	CS	D	1	0	2	Walking with caregiver (W03^f^, W04^g^)	Extrinsic obstacle (other resident in wheelchair in the pathway) Impulsivity and aberrant behavior	0
	#4	CS	D	0	0	2	Transfer sit-to-stand (W07^e^)	No extrinsic factor identified	0
	#5	CS	N	0	0	2	Transfer sit-to-stand (W07^e^)	No extrinsic factor identified No anticipation/awareness of her purse blocking her leg	1
	#6	CS	D	0	0	2	Slipping from chair (W07^e^)	No extrinsic factor identified	0
	#7	CS	D	0	0	3	Walking (W01^h^)	No extrinsic factor identified Distraction	0
	#8	CS	D	0	0	2	Transfer sit-to-stand (W07^e^)	No extrinsic factor identified No anticipation/awareness of her purse blocking her valid hand	0
	#9	CS	D	0	0	2	Transfer sit-to-stand (W07^e^)	No extrinsic factor identified No anticipation/awareness of her purse blocking her valid hand	1
	#10	CS	D	1	0	3	Moving with wheelchair (W05^i^)	No extrinsic factor identified Impulsivity	0
**S2 M**									
	#1	BR^j^	D	1	0	3	Transfer stand-to-sit while dressing (W06^k^)	Environmental hazard (messy bed) Environmental distractor (door open-closed) Poor judgment of the dangerous situation (dual tasking, no appraisal of distance, inappropriate sitting)	0
**S3 M**									
	#1	BR	N	0	0	3	Walking/loss of support (W03^f^, W06^k^)	Environmental stressor (subject pushed from other resident’s bed) Inappropriate use of mobility aid (rollator) Aberrant behavior/confusion	0
	#2	BR	D	0	0	3	Transfer stand-to-sit (W08^l^, W06^k^)	Environmental hazard (grabbing clothes on the floor) Poor awareness of his deficits and of the dangerous situation	0
	#3	BR	D	1	0	4	Transfer sit-to-stand (W06^k^)	Environmental hazard (slippery bed blanket/messy bed) No anticipation of the dangerous situation No call for assistance (3 hours—time spent lying on the ground) Confusion	0
**S4 M**									
	#1	BR	N	1	1	3	Transfer stand-to-sit (W06^k^)	Environmental hazard (slippery bed sheet/messy bed and poor lighting) Lack of judgment Inappropriate transfer strategy and use of rollator Poor appraisal of distance	1
**S5 M**									
	#1	BR	N	0	0	2	Transfer lay-to-sit (W06^k^)	Environmental hazard (slippery bed sheet/messy bed) Poor awareness of deficits Impulsivity	0

^a^F indicates female and M indicates male.

^b^D indicates day and N indicates night.

^c^International Classification of Diseases, Tenth Edition (ICD-10).

^d^CS: common space.

^e^W07: fall involving chair.

^f^W03: other fall on same level due to collision with, or pushing by, another person.

^g^W04: fall while being carried or supported by other persons.

^h^W01: fall on same level from slipping, tripping, and stumbling.

^i^W05: fall involving wheelchair.

^j^BR: bedroom.

^k^W06: fall involving bed.

^l^W08: fall involving other furniture.

## Discussion

### Principal Findings

This observational study brings evidence that continuous video monitoring and video review of falls of residents in a memory care facility can support best quality of care. It was found in this pilot study that continuous video monitoring in common spaces and private bedrooms of such care facility and fall review were both feasible and acceptable by facility staff after a certain adoption period. Although these preliminary results need to be confirmed with a larger number of facilities and a larger sample of participants and fall cases in future studies, fall review appears as a valuable health care procedure that might contribute to improved safety in residents and yield better quality of care in facility practice. Fall review provides a unique access to the unpredictable unwitnessed history of a fall, thus supporting screening for the severity of the fall and fall-related injury at the acute phase. Video replay might also allow for secondary prevention in high-risk multi-faller residents with cognitive disorders and, more broadly, for updated facility care policies and preventative actions regarding the living environment of all residents.

Although the fall rate is quite high in long-term care facilities [30], the difficulty to capture real-world fall data is now widely acknowledged and the research in the field is scarce [28]. To the best of our knowledge, this study is the first to report on video recording and review in both common and private spaces (ie private bedrooms) of a health care facility. Although another group in Canada has been evaluating a larger sample of 227 falls in two care facilities in common spaces only [30], a recent study investigating administrative records about 70,000 falls in 528 German long-term care facilities reported that 75% of falls occur in residents’ rooms [38]. Bearing in mind the major issue of privacy and intrusiveness of health technology in private spaces [39], the results of this study point out the advantages of investigating falling patterns in private bedrooms where most of silent and severe falls were captured (if the multi-faller woman [subject 1] would be excluded). Although the Hawthorne effect has been described (ie, individuals modifying their behavior in response to their awareness of being observed) [40], facility staff did not report orally any such secondary effects in residents or in professional caregivers. However, it must be noted that only falls were of interest in this study, and other behaviors and behavior changes related to the presence of video recording were not studied here. The interviews showed that after an adoption period, facility staff began to incorporate the video review in their traditional care practice during regular staff care meetings of the final month. Implementation of video review triggered off policy changes and practice improvement (additional safety rounds for high at-risk residents and environmental changes when situational factors had been identified as key contributors of falls), which might account for the drop in fall rate during the final month of the study. In that perspective, these preliminary results contrast with other health technologies, such as bed alarms, that did not show a decrease in the incidence of falls in hospitalized patients [27]. Interestingly, two-thirds of falls occurred during transfers of any type that confirms [30] that professional caregivers should pay more attention to dangerous transition activity periods. This also raises the question of the correct benefit-risk trade-off, whether to let at-risk residents stay active independently (but then lowering safety) or be overly protective by restricting their activities (but then precipitating their loss of autonomy) [41]. Regarding repeated falls of subject S1, a wheelchair was introduced by facility staff at some time point during the study, probably because of her repeated falls. Whether the introduction of the wheelchair was related to the video monitoring remained unknown. However, this preventative strategy was not fully successful as it appeared that she fell from her wheelchair also (fall #10), most probably in relation to her neurological disorders. Finally, although not observed in this study, environmental modifications such as compliant flooring [42] or usage of video to train caregivers about at-risk situations [23] have also been reported to manage and prevent falls and ultimately enhance quality of care in care facilities.

The video footage gave access to unrivaled data that were explored from a multidisciplinary perspective, thanks to the combination of the information gathered during the meetings with the facility staff and the analyses of the videos carried out by the researchers. A first finding is that rapid postfall review provides a unique access to the ever-unpredictable “unwitnessed” hidden and silent event of the fall. Access to the natural history of the fall is all the more challenging because individuals suffering from cognitive impairment including memory loss are usually unable to recall the fall [43]. Video capture provides an exclusive support to diagnose the fall (in case of autonomous lift from the floor; Figure 7), to investigate fall-related injuries (given fall direction and body impact), and for grading the severity of the injury requiring further paramedical and/or medical examinations (high-speed falls with traumatic injury for instance; Figures 5 and 6). Traumatic brain injury in particular is one of the most severe and frequent related injury (with an estimated frequency of 33% to 37% in falls [44]). Although the video review was not used in real time in this pilot study, the use of the 4-point Hopkins Falls Grading Scale [35] suggests that video could be a rapid and efficient screening tool to categorize residents requiring either direct emergency referral or in-facility nursing checking or even just regular routine supervision. Furthermore, severity screening and fall anamnesis could be used both in-place and remotely to support decision making of health professionals. Although prior studies have investigated in detail the benefits of video capture for understanding the biomechanical features of falls [30-33], this study suggests that such an assistive health technology tool could efficiently complement (not replace) existing routine care [45] in some care settings. If integrated into a tele-care loop, video reviews of falls clearly offer benefits for patients in terms of better diagnoses of fall-related injuries [45]. Although not documented yet in terms of cost-effectiveness analysis, such a technology-assisted care raises major public health and economics issues in terms of cost savings and better care organization in nursing facilities: more efficient allocation of human resources within facilities could be further discussed, and unnecessary external referral to the emergency unit could be spared, or, reverse, more fall-related comorbidities could be cared for early [2,16]. Given the aging population, the high cost of Alzheimer disease (the single most expensive disease in the United States with an estimated yearly US $236 billion direct costs and US $221 billion indirect costs [5]), and the growing number of care needs in memory care facilities, video-enabled technology avoiding time-consuming and costly black-sighted exploration such as total body scan in case of postfall confusion, as well as hospitalizations and unanticipated comorbidities, could be of great interest for health regulators [46].

This study makes it also challenging to analyze the complex multifactorial falling patterns through video in the particular perspective of cognitively impaired older adults. Factors that contribute to the risk of falls in patients have traditionally been classified as intrinsic (individual predisposition), extrinsic (environmental hazard), and situational (related to the activity being done) [47-51]. These factors have to be addressed to maximize primary and secondary prevention of falls, a major public health and clinical issue (PubMed identifies 5048 papers published on [fall] in 2016), despite a substantial lack of standardization in fall management [16]. Although the impact of environmental modifications on falls and fall-related injuries has been difficult to measure [17] **,** the findings of this study reinforce recent major studies that showed that home-safety assessment and modifications impacting extrinsic factors reduce falls by 19% to 26% [20,52]. The personalized room-safety modifications (ie, tailored interventions aiming at modifying extrinsic risk factors in the bedrooms of residents) that the facility board reported after video review could account for the drop in fall rate observed in the last month of the study. However, it must be acknowledged that the persistence of the low fall rate over time was not measured (as the study was over after 3 months) and that residents’ turnover might affect fall rate differently in the future. Nevertheless, environmental modifications, one of the four prevention pillars identified by the World Health Organization to prevent falls aside from preventative actions targeting behavioral, biological, and socioeconomic risk factors [21], should be now systematically addressed in health care settings [22,15].

As previously stated, a fall is usually multifactorial and happens as a result of a complex interaction between the individual and his or her living environment [34]. An additional interesting question raised by this research is to find out whether part of cognitive processing and cognitive-behavioral dysfunction before, during, and after the fall can be observed through video review and thus be potentially addressed in the perspective of secondary prevention. Although studies about falls in dementia are numerous, only few authors approached the cognitive component during the falling process and rather recently [50-57]. The St Louis OASIS study classification allocated three out the 24 items to cognition (global cognitive impairment, visual-perceptual impairment [ie, misperceiving the environment], and distraction) and categorized them into the intrinsic factor class. The main research group in the field of video monitoring of falls [17] investigated the falling process in various population, including elderly with and without dementia, and put its focus mainly on the level of functional and biomechanical features; this group studied in detail fall stages (initiation, descent, and impact) and landing configuration and fall direction and addressed causes in terms of cause of imbalance, activity at time of the fall, gait, balance, and motor and functional dysfunction. This group briefly discussed in one of its papers the fact that the cognitive status and psychological state could be a contributing factor to falls [51]. Although no audio was recorded (that could give us more data on behavioral-cognitive disorders), the existing data suggest that part of cognitive-behavioral dysfunction as a particular risk factor can be observed on video footage. The video review might suggest that cognitive-behavioral dysfunction (and executive dysfunction in particular), a major contributor of fall in dementia [4,53,54], can also be partially observed in some cases, where lack of judgment and poor awareness of the danger, poor appraisal of self-deficits and of distances, impulsivity, inattention, and over-reactivity to external distractors in the environment are observable. However, these assumptions about neurocognitive observations need to be confirmed over a larger number of video recordings of falls and should include multiple raters’ assessment in the future. Also, other extrinsic and intrinsic factors such as the lighting variation or the fatigue of the individuals should be taken into account as part of the multiple factors that might account for the fall. This proposed holistic framework that includes video observation of cognitive-behavioral dysfunction within its interaction with the living and social environment of individuals might reinforce recent findings documenting that executive dysfunction is strongly associated with multiple falls [53,55] and that cognitive training (apart from motor and gait training) is an underexplored but resourceful approach in reducing falls [56,57]. More attention when reviewing videos of falls should be paid in the future to the complex interaction between cognitive-behavioral responses and the social-contextual stimuli of the living environment just before the fall.

### Limitations and Recommendations

This study needs to be replicated and results confirmed over a larger sample size of individuals and memory care facilities and over a longer period of time to control for size effect, to measure long-lasting effects, and to allow for meaningful examination of the relation between decrease in fall rate and the proposed intervention. Recommendation for future research include (1) upgrading computational deep-learning algorithms to provide an automated diagnosis (or assumption) of real-time fall, as well as an at-risk screening scale estimating the fall risk in every resident, thanks to an automatized set of video-based biomarkers; (2) measuring time spent lying on the floor (time-to-event between the fall and caregiver intervention); (3) conduction of further studies (if possible randomized) comparing conventional care with real-time utilization of an interactive assistive video diagnostic of falls; (4) proposing a cost-effectiveness analysis of using such technology in memory care facilities; (5) conducting interviews within focus groups using medical anthropology approaches to get a deeper understanding about professional caregivers’ perspective on the video monitoring; (6) increasing knowledge about fall epidemiology and falling patterns regarding cognitive functioning of the individuals in particular (including distinct pathologies such as Alzheimer disease, Parkinson disease, Lewy body disease, and frontotemporal dementia); and (7) deploying and testing the device in other settings such as individual homes.

### Conclusions

Falls and fall-related injuries are frequent and potentially preventable causes of morbidity, functional decline, and increased health care use and mortality among individuals suffering from Alzheimer disease and related disorders. The findings of this study highlight the potential of video-monitoring deployment to support fall diagnostic and fall-related injuries and suggest that video review can have a positive impact on quality of care in memory care facilities. Given the growing demand for assisted living in elderly and persons with dementia, video monitoring appears as a promising assistive tool to support health care organizations and possibly complement existing conventional care for both detection and prevention of falls. But more data are needed to validate that the fall rate in managed care facilities can be reduced and safer care provided through interactive video review of falls.

## Abbreviations

CDSS-CCLD: California Department of Social Services Community Care Licensing Division
ICD-10: International Classification of Disease, Tenth Edition
NAS: network attached storage
SD: standard deviation
